# Complicated grief and related factors among nursing staff during the Covid-19 pandemic: a cross-sectional study

**DOI:** 10.1186/s12888-023-04562-w

**Published:** 2023-01-26

**Authors:** Farnaz Rahmani, Mina Hosseinzadeh, Leila Gholizadeh

**Affiliations:** 1 Social Determinants of Health Research Center, Tabriz University of Medical Sciences, Tabriz, Iran; 2 Department of Community Health Nursing, Nursing and Midwifery Faculty, Tabriz University of Medical Sciences, Tabriz, Iran; 3 Faculty of Health, University of Technology, Sydney, NSW Australia

**Keywords:** Grief, Nursing Staff, Covid-19

## Abstract

**Background:**

The challenging working conditions during the Covid-19 pandemic created a perfect storm that can seriously impact nurses' physical and psychological well-being. Our study aimed to investigate complicated grief and its related factors among nursing staff during the Covid-19 pandemic.

**Methods:**

This is a cross-sectional study. The participants comprised 375 nurses selected by the convenience sampling method from designated wards for patients with Covid-19 in 3 hospitals in Tabriz, Iran. Participants completed a survey containing demographic and clinical questions, the Inventory of Complicated Grief, and the Multidimensional Scale of Perceived Social Support. Multiple regression analysis was used to identify the associates of nurses' grief. The STROBE guidelines were followed in reporting the study's findings.

**Results:**

A significant proportion of participants (57.6%) were found to be suffering from complicated grief. Gender, educational background, type of ward, type of nursing role, type of working shift, years of nursing work experience, and experience working in the Covid-19 settings remained the significant associates of nurses' grief in the regression analysis.

**Conclusion:**

Due to frequent exposure to patients' deaths, healthcare providers are at increased risk of suffering from complicated grief during the Covid-19 and post-pandemic. If it remains unresolved, complicated grief can result in significant health problems and the experience of burnout among nurses. Governments, health authorities, and nursing managers should support nurses who work in Covid-19 settings to reduce the adverse impact of the pandemic on nurses' health and well-being.

## Introduction

The Covid-19 pandemic presents unprecedented challenges to all sectors of societies, in particular health care systems [1]. Nurses play a critical role in care provision to patients with Covid-19. As the impact of Covid-19 on individuals and healthcare systems began to manifest, nurses found themselves in situations never seen before, frequently working long hours within uncertain circumstances, limited access to personal protective equipment, and evolving guidance on how to care for patients with coronavirus disease [2].

Working in such conditions, being exposed to a deadly virus, working long shifts with a heavy workload, and being frequently involved in end-of-life care, can adversely affect the mental health of nurses [3, 4]. Traditionally, the nurse is either the proxy for a family that cannot be present at the patient's bedside or supports the patient and the family as they transition through the end-of-life journey [5]. While Covid-19 patients are isolated without access to visitors, nurses are frequently involved in providing emotional support to patients and their families [6]. The challenging conditions and new ways of working have created a perfect storm that can seriously impact nurses' physical and psychological well-being [7]. Results of a review study suggested that during the recent pandemic, healthcare workers experienced a high level of trauma-related stress, ranging from 7.4% to 35%. Trauma-related stress was notably higher among nurses, frontline health professionals, female healthcare workers, and those who experienced some physical symptoms [8]. In addition, a systematic review revealed that post-traumatic stress was more prevalent among young healthcare workers, those with less work experience and heavy workloads, healthcare workers working in unsafe settings, and those lacking adequate training and sufficient social support [9]. Among the factors contributing to nurses' burnout syndrome were prolonged night shifts, years of experience, and exposure to traumatic events [10].

Overall, the prevalence of mental health issues augmented during the Covid pandemic. A systematic review that focused on the prevalence of mental health issues among nurses during the Covid-19 pandemic reported that 37%, 35%, and 43% of nurses suffered from anxiety, depression, and poor sleep quality during the pandemic [11]. A large study in China reported the prevalence of anxiety, depression, and poor sleep quality to be 35.1%, 20.1%, and 18.2%, respectively, among the public during the pandemic. Compared with other occupations, healthcare workers experienced poorer sleep quality [12]. A systematic review of workplace violence during the Covid-19 pandemic concluded that nurses may be exposed to psychological workplace violence as they provide care to patients with life-threatening Covid-19 infections, which may negatively affect their mental health [13].

Frequent exposure to patients' deaths leads to feelings of helplessness and despair among nurses [6]. The novelty of Covid-19 and nurses' inability to prevent patients' death negatively impact their mental health [14]. Healthcare professionals may be saddened by the deaths of young patients or patients who could not be treated with medical interventions [15, 16]. Working with severely ill patients and frequent exposure to patients' deaths can increase work-related stress among nurses and lead to a profound sense of grief [17, 18]. Nurses may need to be alone and withdraw from daily activities for short periods to recover from stressful situations they experience at work [19].

Grief is a healthy process that helps an individual to adapt to loss. However, the response of healthcare providers, especially nurses, to patients' deaths during the Covid-19 pandemic may be different due to their multiple exposures to patients' deaths. Some healthcare workers may ignore the feeling of grief and use strategies, such as avoidance or submission, to continue to work [20, 21]. An inappropriate or incomplete grief process can result in pathologic or complicated grief leading to maladaptive symptoms [22, 23]. The grieving process takes time and needs the support of friends and colleagues [24]. During the covid-19 pandemic, nurses may not have enough time to process their grief leading to unresolved grief, mental health disorders, and burnout [25]. Feeling a sense of responsibility and obligation to protect patients' lives can also increase nurses' risk of complicated grief [26].

In the era of the Covid-19 pandemic, nurses are expected to work long hours, accept additional shifts, and bear heavy workloads due to the increased number of hospitalizations and staff shortages. Consequently, they may not have enough time to vent their emotions and express their psychological reactions to patients’ deaths [26]. This situation can be more complicated when patients quickly transition from ‘‘being ill’’ to ‘‘dead” [27]. Although nurses are more vulnerable to grief in the era of the pandemic, referred to as the 'tsunami of death' [19], little work has been done to understand nurses' grief process and the factors that contribute to it [20, 28]. This study aimed to investigate grief patterns in healthcare providers working in Covid-19 wards and its related factors. This understanding is vital to provide evidence to inform supportive interventions to maintain the health and well-being of nurses during the recent pandemic and other possible future pandemics.

## Methods

### Design

We used a descriptive correlational study design to investigate grief patterns and related factors among nurses working in designated Covid-19 wards.

### Participants and settings

The target population for the current study was nurses working full-time at 3 centers designated for Covid-19 patients in Tabriz, Iran. The sample size estimation was calculated using the results of a pilot study on 30 nurses using the results of the pilot study (Confidence Interval (CI) of 95%, test power of 80%, α = 0.05, *r* = 0.36), but given the availability of subjects and the possible non-response rate of 15%, the required sample size was ultimately raised to 425. The sampling was conducted between February 2022 and July 2022.

The survey link was distributed via social platforms, including WhatsApp, Telegram, and Instagram applications, and nurses who met the following criteria were invited to participate in the study: 1) nurses who were working at a Covid -19 ward, and 2) who had faced a patient's death due to Covid -19 more than six months ago, 3) having no history of psychiatric illness, according to self-reports. Participants who missed answering more than 50% of the questions on each scale were excluded.

### Instruments

The data were collected using the Inventory of Complicated Grief (ICG) [29] and the Multidimensional Scale of Perceived Social Support (MSPSS) [30]. These scales are free for public access. The ICG was developed by Prigerson et al. (1995) to assess the symptoms of complicated or pathological grief. It is a 19-item self-report measure, which uses a five-point Likert-type scale, with response options ranging from 0 (never) to 4 (always). The developers recommended a cutoff score of > 25 to distinguish between uncomplicated and complicated grief patterns [29]. The scale has proven transcultural validity, with internal reliability (as measured by Cronbach's alpha) ranging between 0.87 and 0.94 [31, 32]. In the current study, the researchers prepared a Persian version of the scale by adopting the translation and back-translation procedure [33]. The translated scale was pilot-tested concerning its relevance and clarity with 30 nurses who experienced the loss of a patient with Covid-19 more than six mounts ago. The reliability of ICG (Persian form) was confirmed using test–retest (*r* = 0.81).

The MSPSS is a 12-item measure of perceived adequacy of social support from three sources: family (items 3, 4, 8, and 11), friends (items 6, 7, 9, and 12), and significant other (items 1, 2, 5, and 10). The tool uses a seven-point Likert scale ranging from 1 (very strongly disagree) to 7 (very strongly agree). A higher score indicates greater social support perceived by an individual; the total score can range between 12 and 84. The MSPSS has been used widely in different settings [34–36]. In the present study, Cronbach's alpha coefficient and the intra-class correlation coefficient (ICC) for the MSPSS were 0.89 and 0.92, respectively.

In addition, we collected data on the socio-demographics of participants, including age, gender, educational level, marital status, and income, as well as work-related factors, including the type of nursing role, nursing work experience, work experience in Covid-19 wards, type of work shift, and type of ward. An expert panel, including 12 faculty members from Tabriz University of Medical Sciences, confirmed the face and content validity of the survey package.

### Data analysis

Data were analyzed using the Statistical Program for the Social Sciences (SPSS) version 13.0 for Windows. Descriptive statistics were computed for all variables, including frequencies, means, and standard deviations (SD). The Kolmogorov- Smirnov test was used to examine data distribution. The variables of grief and social support were normally distributed with skewness and kurtosis indices less than ± 2 [37]. Relationships between grief and social support and other socio-demographic and work-related variables were assessed using the Pearson correlation coefficient, independent t-tests, ANOVA tests, and multiple regression analysis. Statistical significance for all tests was set at *p* < 0.05.

## Results

### Demographic characteristics

Of 425 participants, 50 respondents (11.7%) did not answer more than 50% of the questions on each scale and were excluded from the analysis (a response rate of 88.3%). Participants were primarily women (65.8%), with a mean age of 31.9 ± 8.3 years. They had a mean nursing work experience of 9.7 ± 3.1 years and experience in COVID-19 wards of 2.3 ± 0.7 years. The sample characteristics are presented in Table 1.

**Table 1:** Distribution of socio-demographic characteristics and work-related factors

Variables		n (%)
Age (years)	Mean (SD)	31.9 ± 8.3
	≤ 35	228 (60.1)
	> 35	147 (39.9)
Gender	Male	137 (36.5)
	Female	238 (63.5)
Marital Status	Married	203 (54.1)
	Single	172 (45.9)
Income	Income less than expenditure	22 (5.8)
	Income equivalent to expenditure	274 (73.1)
	Income more than expenditure	79 (21.1)
Educational background	Bachelor’s degree	273 (72.8)
	Master's degree	102 (27.2)
Type of ward	Covid-19 ICU	144 (38.4)
	General ward for Covid-19	231 (61.6)
Nursing work experience	< 10 years	196 (52.7)
	≥ 10 years	179 (47.3)
Work experience in COVID-19 settings	< 2 years	143 (38.1)
	≥ 2 years	232 (61.9)
Type of nursing role	A floor nurse	293 (78.1)
	Nurse manager (head nurse/clinical nurse supervisor)	82 (21.9)
Type of shift	Fixed	160 (43.2)
	Rotating	215 (57.3)

The mean (SD) of grief scores was 29.4 ± 7.1, and 57.6% of participants experienced complicated grief. The mean (SD) of social support scores was 23.7 ± 6.2. The mean (SD) of social support categorizes is presented in Table 2.

**Table 2:** Distribution of pattern of grief and perceived social support among participants

Variables		n (%)
The pattern of grief (total score) [Range: 0–76)	Mean (SD	29.4 ± 7.1
The pattern of grief (categories)	Uncomplicated	159 (42.4)
	Complicated	216 (57.6)
Social support (total score) [Range: 12–84]	Mean (SD)	23.7 ± 6.2
Social support (categories) [Range score: 7–28]		
Family support		10.3 ± 3.1
Friends support		12.6 ± 3.4
Significant others support		12.9 ± 3.5

The Pearson r correlation coefficient showed a statistically significant negative mode correlation between scores of grief and social support (*r* =  − 0.49, *p* < 0.001) and its different dimensions (friends support: *r* =  − 0.41, *p* < 0.001; family support: *r* =  − 0.47, *p* < 0.001; and significant others (*r* =  − 0.43, *p* < 0.001), Table 3.

**Table 3:** Association between patterns of grief and social support

Variables	Complicated grief	Uncomplicated grief	Total Greif (The ICG scores)	Friends support	Family support	Significant other support	Social Support (The MSPSS scores)
Complicated	-	-0.19*	0.29**	-0.34**	-0.41***	-0.47***	-0.48***
Uncomplicated		-	0.26**	0.29*	0.23*	0.34**	0.32*
The grief scores			-	-0.41***	-0.47***	-0.43***	-0.49***
Friend social support				-	0.24*	0.19*	0.21*
Family social support					-	-0.12*	0.21*
Significant other support						-	0.17*
The social support scores							-

^*^p < 0.05, **p < 0.01, ***p < 0.001

The mean of grief scores was higher among females (29.4 ± 8.7, *p* < 0.001), single (27.2 ± 6.1, *p* < 0.001), and floor nurses (38.3 ± 10.6, *p* < 0.001), and those who had bachelor’s degree (29.7 ± 6.8, *p* < 0.001) and lower income than expenditure (31.6 ± 8.4, *p* < 0.001). Nurses who were working in a Covid-19 intensive care unit (ICU) (34.2 ± 9.1, *p* < 0.001), had nursing work experience > 10 years (29.4 ± 6.7, *p* < 0.001), work experience in Covid-19 wards > 2 years (35.7 ± 9.6, *p* < 0.001) were more likely to experience pathologic/ complicated grief (Table 4).

**Table 4:** Mean grief scores according to socio-demographic and work-related variables

Variables		Complicated grief n (%)	Uncomplicated grief n (%)	Mean ± SD of the grief scores	
Age (years)	≤ 35	161 (70.6)	67 (29.4)	27.6 ± 6.9	*r* = -0.07 *p* = 0.19
	> 35	98 (66.7)	49 (33.3)	26.2 ± 6.4	
Gender	Male	94 (68.6)	43 (31.4)	25.6 ± 7.3	t = 2.47 *p* < 0.001
	Female	181 (76.1)	57 (23.9)	29.4 ± 8.7	
Marital Status	Married	79 (38.9)	124 (61.1)	25.6 ± 5.7	t = 2.76 *p* < 0.001
	Single	117 (68.0)	55 (32.0)	27.2 ± 6.1	
Income	Income less than expenditure	15 (68.2)	7 (31.8)	31.6 ± 8.4	F = 3.44 *p* < 0.001
	Income equivalent to expenditure	214 (78.1)	60 (1.9)	27.3 ± 6.3	
	Income more than expenditure	23 (29.1)	56 (70.9)	24.1 ± 5.1	
Type of ward	Covid-19 ICU	112 (77.8)	32 (22.2)	34.2 ± 9.1	t = 2.63 *p* < 0.001
	General ward for Covid-19	159 (68.8)	72 (31.2)	29.3 ± 8.3	
Educational background	Bachelor’s degree	186 (66.7)	91 (33.3)	29.7 ± 6.8	t = 2.36 *p* < 0.001
	Master's degree	69 (67.6)	33 (32.3)	26.4 ± 5.7	
Nursing work experience	< 10 years	83 (42.4)	113 (57.6)	25.3 ± 5.4	t = 3.17 *p* < 0.0001
	> 10 years	108 (60.3)	71 (39.7)	29.4 ± 6.7	
Work experience in Covid-19 settings	< 2 years	54 (37.8)	89 (62.2)	25.8 ± 5.6	t = 3.64 *p* < 0.001
	> 2 years	153 (68.5)	79 (31.5)	35.7 ± 9.6	
Type of nursing role	A floor nurse	217 (74.1)	76 25.9)	31.2 ± 8.3	t = 2.33 *p* < 0.0001
	Manager/educator	47 (57.3)	35 (42.7)	26.2 ± 6.4	
Type of shift	Fixed	112 (70.0)	48 (30.0)	27.2 ± 5.9	t = 3.13 *p* < 0.0001
	Rotating	68 (31.6)	147 (68.4)	25.4 ± 5.2	

r: Pearson correlation coefficient test; t: Independent t-test; F: AVOVA test

Variables with *p* < 0.05 in univariate analysis were entered into the multiple linear regression model (gender, marital status, income, type of ward, years of nursing work experience, years of experience in Covid-19 wards, type of nursing role) and their relationship with grief, while controlling for the effect of other variables, was determined. The results of multiple linear regression analysis showed that considering the confounding variables, the relationship between the social support score and the grief score was significant (β = -3.473, *P* < 0.001), (Table 5). Based on this result, with a one-point increase in the nurses’ social support score, their grief score decreased by 3.473. Female nurses (vs. male) had a higher grief score (β = 3.372; *P* = 0.029). Regarding the educational background, nurses who studied up to bachelor’s degrees had higher grief scores than nurses who studied up to master’s degrees (β = -3.743; *P* = 0.031). Those who worked in Covid-19 ICU (vs. those who worked in the general ward for Covid-19) had a higher grief score (β = 4.624; *P* < 0.001). There was a higher grief score among nurses with 10 or more years of experience than other nurses (β = 2.328; *P* < 0.001). Also, nurses whose work experiences in Covid-19 wards were more than two years had a higher grief score than other nurses (β = 3.231, *P* < 0.001). Floor nurses (compared to nurse managers) and nurses with a fixed shift type (compared to rotating shifts) had higher scores for grief (β = 2.311; *P* < 0.001 and β = 3.273; *P* < 0.001, respectively). These variables were determinants of the grief score and explained 59.3% (Adjusted R-squared = 59.3%) of the variance (variability) of grief (Table 5).

**Table 5:** Predictors of complicated grief in multiple linear regression analysis

Complicated grief	Unstandardized Coefficients (β)	Std. Error	Standardized Coefficients (β)	*t*	*p-value*	*R*^2^
Constant	-0.4641	6.28		-0.674	0.712	0.593
**Gender (Reference: male)**						
Female	3.372	2.94	0.181	2.543	0.029	
**Marital status (Reference: married)**						
Single	1.391	3.77	0.141	2.432	0.731	
**Income (Reference: Income more than expenditure)**						
Income less than expenditure	3.725	4.21	0.179	4.239	0.311	
Income equivalent to expenditure	-3.349	4.11	0.181	4.126	0.429	
**Educational background (Reference: Master)**						
Bachelor’s degree	3.743	2.42	0.175	2.417	0.031	
**Type of ward (Reference: General ward for Covid-19)**						
Covid-19 ICU	4.624	2.36	0.167	2.329	< 0.001	
**Nursing work experience (Reference: < 10 years)**						
≥ 10 years	2.328	3.45	0.168	2.212	< 0.001	
**Work experience in Covid-19 settings (Reference: < 2 years)**						
≥ 2 years	3.231	4.17	0.154	2.678	< 0.001	
**Type of nursing role (Reference: Master: Nurse manager)**						
A floor nurse	2.311	3.73	0.171	4.117	< 0.001	
**Type of shift (Reference: Rotating)**						
Fixed	3.273	2.74	0.192	3.241	< 0.001	
**Social support**	-3.473	2.17	-0.164	2.417	< 0.001	

## Discussion

A considerable percentage of nurses (57.6%) working on Covid-19 wards experienced a complicated pattern of grief, more likely due to frequent exposure to patients' deaths. Previous studies have consistently shown that nurses who experience multiple deaths of patients under their care may not process their grief and move on due to a lack of time and support during the Covid -19 pandemic [38, 39]. In addition, sudden deaths, painful disease processes, hesitancy about the available medical treatment for coronavirus [40, 41], and feeling obligations to protect patients' lives increase the risk of complicated grief among nurses [42]. It is emotionally challenging for nurses when they cannot alleviate a dying patient's suffering, as it triggers feelings of professional helplessness and weaknesses [43].

Compared to previous studies [44, 45], this study suggests that nurses who work in Covid -19 wards working with patients with Covid-19 infection experienced an alarmingly higher level of complicated grief over the death of the patients. This finding may be explained more by the higher rate of deaths from Covid-19 in Iran (3.5% deaths of the overall total patients confirmed cases of Covid -19 until Mar 20 2022) [46], which could compromise the nurse's mental health due to their multiple exposures to death [21]. Although patient death is a common part of clinical nursing [47], frequent exposure to patients’ death increase the probability of complicated or chronic grief among nurses [19] and decreases the quality of care delivered to patients [43].

A systematic review reported that pediatric oncology nurses' level of chronic and unresolved grief and other psychological hazards is moderate to severe [44]. The high level of complicated grief in our participants calls for urgent attention from health authorities in Iran to implement effective interventions to protect the health and well-being of nurses as the largest healthcare frontline workforce.

In addition, nurses in the current study received a suboptimal level of support from friends, family, and significant others, a factor that contributed to their complicated grief. Overall, Covid-19 quarantines, lockdowns, and social distancing reduced opportunities for socializing and, therefore, the support from families and friends [48]. Lorenzo and Carrisi (2020) reported that fears of Covid -19 transmission isolated nurses from support providers [49]. Social support helps nurses alleviate their stress and prevent burnout [50].

In the regression analysis, nurses who were female, studied up to bachelor's degree, and perceived lower social supports were more likely to experience complicated grief. Consistent with our findings, previous studies mostly show greater grief responses to patient death among female health providers than men [51–53]. Longitudinal research has revealed that women react differently to a loss than men [54]. They suffer significantly higher levels of depression and anxiety while suddenly facing their loved one's death [51, 55]. Thus, female nurses may be more vulnerable to long-term consequences of working in high-mortality and high-stress environments such as ICU and providing care to critically ill patients such as patients with Covid-19. As such, female healthcare workers may experience the chronic impacts of crises such as the Covid -19 pandemic more than their male counterparts [55].

Nurses with a bachelor’s degree were more likely to experience complicated grief than those with higher degrees. This could be because nursing with bachelor's degrees are more likely to work as floor nurses and therefore be exposed to patients' deaths than those with higher nursing degrees who often take managerial roles. Further, a higher educational level has been shown to positively affect nurses' mental health and workability [56]. Nurses with a higher education level demonstrate a better mental capacity to withstand workplace stress and better resistance to hazards at the workplace [57].

Nurses who perceived lower social support were more likely to experience complicated grief. The beneficiary effect of social support on coping with deaths has been widely reported [58]. Loneliness and inadequate social support increase the risk of mental health issues for grievers [59].

In addition, some work-related factors were found to predict the experience of complicated grief among nurses. Nurses who worked as floor nurses and in a Covid-19 ICU were more likely to experience complicated grief. These results are expected as nurses who work directly with patients with Covid-19 or work in an ICU setting experience greater exposure to patient deaths than nurses who work in managerial positions or a medical ward with relatively stable patients. Nurses are expected to care for patients while maintaining a professional relationship, which can be challenging [60]. Nurses are not exempt from the emotional influence of death; they go through the grieving process and experience various emotions and feelings that can chronically affect their health [18]. Providing direct care to a dying patient with a less-known disease, such as Covid -19, and closely witnessing a patient's suffering can profoundly impact nurses' mental health [16]. Therefore, hospital managers and policymakers need to implement workplace health promotion activities such as spirituality-based training, fewer working hours, support, and communication with leadership in order to reduce the hazards associated with work-related stress factors on nurses' mental health caused by the Covid -19 pandemic [61].

Finally, nurses with shorter work experience and long experience in working with covid-19 patients were more likely to experience complicated grief. The studies revealed that nurses with less experience were more vulnerable and exhausted [62]. However, at the same time, nurses with more years of experience could be able to keep a distance and set boundaries in end-of-life care [63]. It seems that the higher the nurses 'work experience, the better their coping skills in dealing with stressful situations such as patient deaths [64].

Nurses caring for Covid-19 patients for more than two years reported a high level of complicated grief. The type and volume of losses a person experiences could impact the grieving process and the likelihood of complicated grief [62, 65]. These findings are expected as nurses who work in covid-19 wards for longer periods are exposed to frequent patient deaths and the subsequent grieving processes, which may remain incomplete due to lack of time, support, and staff shortage [26].

In our study, nurses who worked fixed shifts were more likely to experience complications than nurses who worked rotating shifts. This could be because nurses who worked rotating shifts had better opportunities to interact with colleagues and share their experience of the patient's death with several colleagues. This factor can help them process their grief [66].

The study results have significant implications for nurses, nurse managers, and health authorities. The findings demonstrate some negative health impacts of working with Covid-19 patients for nurses. The findings of this study and other similar studies provide a robust platform to promote the health and well-being of healthcare providers, particularly nurses, during the Covid-19 pandemic and post-pandemic. Nurse Managers and health authorities should consider reducing the working hours of nurses in Covid-19 settings and encourage them to take frequent breaks to allow them time to recover from work-related stresses. Nurses, who work in Covid-19 designated wards, in particular Covid-19 ICUs, should be rotated regularly to have opportunities to recover from stressful experiences, such as frequent exposure to patients 'deaths. Having free or subsidized access to mental health care providers may also help these nurses process their grief after a patient's death positively. A supportive work environment could protect nurses in the face of the high mortality rate of patients with the Covid -19 pandemic [67].

### Study strengths and limitations

Because of the criticality of the pandemic, the special conditions at clinical centers, and the necessity for nurses to work long hours during Covid-19, the grief of nurses may not be given much attention. Therefore, a strength of the study was its consideration of nurses' grief patterns in the face of repeated encounters with patient death. Another strength of the study is its analysis of the factors associated with nurses' complicated grief.

The study was a descriptive correlational study, which produced correlational data that did not support cause-and-effect conclusions. Self-reporting nature of the data was the other limitation of our study, which may affect the accuracy of the collected data. The convenience sampling method, collecting data through social media platforms, and obtaining samples from only 3 centers could be methodological limitations of the study. Therefore, future studies should address these issues.

## Conclusion

This study found that a significant proportion of nurses who worked in Covid-19 settings experienced complicated grief. This can negatively affect nurses' physical and mental health and productivity in the workplace. Subgroups of nurses may be at higher risk, including female nurses, floor nurses, those with a lower nursing degree, and those who work extended periods in covid-19 settings, in particular, Covid-19 ICUs. Governments, health policymakers, and nursing managers should be aware of the deleterious effects of the recent pandemic on nurses' health and well-being. They should seek to reduce the impact by employing strategies such as providing support, reducing working hours, rotating nurses between Covid-19 wards and other wards, and providing support, such as bereavement counseling for those who require this service.

## Data Availability

The datasets generated/analyzed during the current study are not publicly available due to ethical concerns but are available from the corresponding author upon reasonable request. (Ethical committee of Tabriz University of Medical Science has restrictions about the availability of data).

## References

[1] Nie A, Su X, Zhang S, Guan W, Li J (2020). Psychological impact of COVID-19 outbreak on frontline nurses: A cross-sectional survey study. J Clin Nurs.

[2] De Kock JH, Latham HA, Leslie SJ, Grindle M, Munoz S-A, Ellis L (2021). A rapid review of the impact of COVID-19 on the mental health of healthcare workers: implications for supporting psychological well-being. BMC Public Health.

[3] Sharif TJ, Hosseinzadeh M, Mahdavi N, Areshtanab HN, Dickens GL (2020). Happiness and its Relationship with Job Burnout in Nurses of Educational Hospitals in Tabriz. Iran Int J Community Based Nurs Midwifery.

[4] Wolf LA, Perhats C, Delao AM, Moon MD, Clark PR, Zavotsky KE. "It’s a burden you carry”: describing moral distress in emergency nursing. J Emerg Nurs. 2016;42(1):37–46. 10.1016/j.jen.2015.08.008. 10.1016/j.jen.2015.08.008 26431742

[5] Hagan TL, Xu J, Lopez RP, Bressler T. Nursing’s role in leading palliative care: A call to action. Nurse Educ Today. 2018;61:216–9. 10.1016/j.nedt.2017.11.037. 10.1016/j.nedt.2017.11.037 PMC5859921 29245101

[6] Mendiola B, Gomez C, Furst C, Rasmussen-Winkler J (2021). Facilitating virtual visitation in critical care units during a pandemic. Holist Nurs Pract.

[7] Sampaio F, Sequeira C, Teixeira L (2021). Impact of COVID-19 outbreak on nurses’ mental health: a prospective cohort study. Environ Res..

[8] Benfante A, Di Tella M, Romeo A, Castelli L (2020). Traumatic stress in healthcare workers during COVID-19 pandemic: a review of the immediate impact. Front Psychol.

[9] d’Ettorre G, Ceccarelli G, Santinelli L, Vassalini P, Innocenti GP, Alessandri F (2021). Post-traumatic stress symptoms in healthcare workers dealing with the COVID-19 pandemic: a systematic review. Int J Environ Res Public Health.

[10] Chirico F, Afolabi AA, Ilesanmi OS, Nucera G, Ferrari G, Sacco A (2021). Prevalence, risk factors and prevention of burnout syndrome among healthcare workers: an umbrella review of systematic reviews and meta-analyses. J Health Soc Sci.

[11] Al Maqbali M, Al Sinani M, Al-Lenjawi B (2021). Prevalence of stress, depression, anxiety and sleep disturbance among nurses during the COVID-19 pandemic: A systematic review and meta-analysis. J Psychosom Res..

[12] Huang Y, Zhao N (2020). Generalized anxiety disorder, depressive symptoms and sleep quality during COVID-19 outbreak in China: a web-based cross-sectional survey. Psychiatry Res..

[13] Chirico F, Afolabi A, Ilesanmi O, Nucera G, Ferrari G, Szarpak Ł (2022). Workplace violence against healthcare workers during the COVID-19 pandemic: a systematic review. J Health Soc Sci.

[14] Maben J, Bridges J (2020). Covid-19: Supporting nurses’ psychological and mental health. J Clinic Nurs.

[15] Nia HS, Lehto RH, Ebadi A, Peyrovi H (2016). Death anxiety among nurses and health care professionals: a review article. Int J Community based Nurs Midwifery.

[16] Das S, Singh T, Varma R, Arya YK (2021). Death and mourning process in frontline health care professionals and their families during COVID-19. Front Psychiatry..

[17] Rabow MW, Huang CHS, White-Hammond GE, Tucker RO (2021). Witnesses and victims both: healthcare workers and grief in the time of COVID-19. J Pain Symptom Manage..

[18] Jang SK, Park WH, Kim H-I, Chang SO (2019). Exploring nurses’ end-of-life care for dying patients in the ICU using focus group interviews. Intensive Crit Care Nurs.

[19] Oates JR, Maani-Fogelman PA. Nursing grief and loss. Florida: StatPearls; 2020. 30085531

[20] Betriana F, Kongsuwan W (2019). Nurses’ grief in caring for patients with advanced cancer: a literature review. Songklanagarind J Nurs.

[21] Abdi M (2020). Coronavirus disease 2019 (COVID-19) outbreak in Iran: actions and problems. Infect Control Hosp Epidemiol.

[22] Wallace CL, Wladkowski SP, Gibson A, White P (2020). Grief during the COVID-19 pandemic: considerations for palliative care providers. J Pain Symptom Manage.

[23] Alizadeh A, Khankeh HR, Barati M, Ahmadi Y, Hadian A, Azizi M (2020). Psychological distress among Iranian health-care providers exposed to coronavirus disease 2019 (COVID-19): a qualitative study. BMC Psychiatry.

[24] Rabin L. Understanding the needs of the suddenly bereaved: The significance of social network support. Death Stud. 2011;5(35):467–73. 10.1080/07481187.2010.515464.

[25] Bellanti F, Lo Buglio A, Capuano E, Dobrakowski M, Kasperczyk A, Kasperczyk S (2021). Factors related to nurses’ burnout during the first wave of coronavirus disease-19 in a university hospital in Italy. Int J Environ Res Public Health.

[26] Galehdar N, Kamran A, Toulabi T, Heydari H (2020). Exploring nurses’ experiences of psychological distress during care of patients with COVID-19: a qualitative study. BMC Psychiatry.

[27] Lobb EA, Kristjanson LJ, Aoun SM, Monterosso L, Halkett GK, Davies A (2010). Predictors of complicated grief: a systematic review of empirical studies. Death Stud.

[28] Barnes S, Jordan Z, Broom M (2020). Health professionals’ experiences of grief associated with the death of pediatric patients: a systematic review. JBI Evid Synth.

[29] Prigerson HG, Maciejewski PK, Reynolds CF, Bierhals AJ, Newsom JT, Fasiczka A (1995). Inventory of Complicated Grief: a scale to measure maladaptive symptoms of loss. Psychiatry Res.

[30] Zimet GD, Dahlem NW, Zimet SG, Farley GK (1988). The multidimensional scale of perceived social support. J Pers Assess.

[31] Montano SA, Lewey JH, O’Toole SK, Graves D (2016). Reliability generalization of the Texas revised inventory of grief (TRIG). Death stud.

[32] Alves T, Oliveira M, Lotufo-Neto F (2016). Diagnosis of complicated grief using the Texas Revised Inventory of Grief, Brazilian Portuguese version. J Psychol Clin Psychiatry.

[33] Ozolins U, Hale S, Cheng X, Hyatt A, Schofield P (2020). Translation and back-translation methodology in health research–a critique. Expert Rev Pharmacoecon Outcomes Res.

[34] Dambi JM, Corten L, Chiwaridzo M, Jack H, Mlambo T, Jelsma J (2018). A systematic review of the psychometric properties of the cross-cultural translations and adaptations of the Multidimensional Perceived Social Support Scale (MSPSS). Health Qual Life Outcomes.

[35] Laksmita OD, Chung M-H, Liao Y-M, Chang P-C (2020). Multidimensional Scale of Perceived Social Support in Indonesian adolescent disaster survivors: A psychometric evaluation. PLoS One..

[36] Zimet GD, Powell SS, Farley GK, Werkman S, Berkoff KA (1990). Psychometric characteristics of the multidimensional scale of perceived social support. J Pers Assess.

[37] Kim H-Y (2013). Statistical notes for clinical researchers: assessing normal distribution (2) using skewness and kurtosis. Restor Dent Endod.

[38] Rabow MW, Huang C-HS, White-Hammond GE, Tucker RO (2021). Witnesses and victims both: Healthcare workers and grief in the time of COVID-19. J Pain Symptom Manage..

[39] Clark-Snow RA, Rittenberg C (2021). Oncology nursing supportive care during the COVID-19 pandemic: reality and challenges. Springer.

[40] Eisma MC, Tamminga A, Smid GE, Boelen PA (2021). Acute grief after deaths due to COVID-19, natural causes and unnatural causes: an empirical comparison. J Affect Disord.

[41] Eisma MC, Tamminga A (2020). Grief before and during the COVID-19 pandemic: Multiple group comparisons. J Pain Symptom Manage.

[42] McCallum KJ, Walthall H, Aveyard H, Jackson D (2021). Grief and nursing: Life and death in the pandemic. J Adv Nurs..

[43] Tornøe KA, Danbolt LJ, Kvigne K, Sørlie V (2015). The challenge of consolation: nurses’ experiences with spiritual and existential care for the dying-a phenomenological hermeneutical study. BMC Nurs.

[44] Boyle DA, Bush NJ (2018). Reflections on the emotional hazards of pediatric oncology nursing: Four decades of perspectives and potential. J Pediatr Nurs.

[45] Adwan JZ. Pediatric nurses’ grief experience, burnout and job satisfaction. J Pediatr Nurs. 2014;29(4):329–36. 10.1016/j.pedn.2018.03.007. 10.1016/j.pedn.2014.01.011 24582646

[46] Ahmadi Gohari M, Chegeni M, Haghdoost AA, Mirzaee F, White L, Kostoulas P, Mirzazadeh A, Karamouzian M, Jahani Y, Sharifi H. Excess deaths during the COVID-19 pandemic in Iran. Infect Dis. 2022;54(12):909–17. 10.1080/23744235.2022.2122554. 10.1080/23744235.2022.2122554 36121798

[47] Sutherland R (2019). Focus: death: dying well-informed: the need for better clinical education surrounding facilitating end-of-life conversations. Yale J biol Med.

[48] Hou T, Zhang T, Cai W, Song X, Chen A, Deng G (2020). Social support and mental health among health care workers during Coronavirus Disease 2019 outbreak: a moderated mediation model. Plos one..

[49] Lorenzo D, Carrisi C (2020). COVID-19 exposure risk for family members of healthcare workers: an observational study. Int J Infect Dis.

[50] Sahay S, Wei W (2022). “Everything Is Changing, but I Am Not Alone”: nurses’ perceptions of social support during COVID-19. Sustainability.

[51] Granek L, Krzyzanowska MK, Nakash O, Cohen M, Ariad S, Barbera L (2016). Gender differences in the effect of grief reactions and burnout on emotional distress among clinical oncologists. Cancer.

[52] Redinbaugh EM, Sullivan AM, Block SD, Gadmer NM, Lakoma M, Mitchell AM, et al. Doctors’ emotional reactions to recent death of a patient: cross sectional study of hospital doctors. BMJ. 2003;327(7408):185. 10.1136/bmj.327.7408.185. 10.1136/bmj.327.7408.185 PMC166122 12881257

[53] Cabarkapa S, Nadjidai SE, Murgier J, Ng CH (2020). The psychological impact of COVID-19 and other viral epidemics on frontline healthcare workers and ways to address it: A rapid systematic review. Brain Behav Immun Health..

[54] Stelzer EM, Atkinson C, O’Connor MF, Croft A. Gender differences in grief narrative construction: a myth or reality? Eur J Psychotraumatol. 2019;10(1):1688130. 10.1080/20008198.2019.1688130. 10.1080/20008198.2019.1688130 PMC6882469 31807234

[55] Morgan R, Tan H-L, Oveisi N, Memmott C, Korzuchowski A, Hawkins K, et al. Women healthcare workers’ experiences during COVID-19 and other crises: a scoping review. Int J Nurs Stud. 2022;4:100066. 10.1016/j.ijnsa.2022.100066. 10.1016/j.ijnsa.2022.100066 PMC8801061 35128472

[56] Kowalczuk K, Krajewska-Kułak E, Sobolewski M (2020). The effect of subjective perception of work in relation to occupational and demographic factors on the mental health of polish nurses. Front Psychiatry..

[57] Golubic R, Milosevic M, Knezevic B, Mustajbegovic J (2009). Work-related stress, education and work ability among hospital nurses. J Adv Nurs.

[58] Scott HR, Pitman A, Kozhuharova P, Lloyd-Evans B (2020). A systematic review of studies describing the influence of informal social support on psychological well-being in people bereaved by sudden or violent causes of death. BMC Psychiatry.

[59] Cacciatore J, Thieleman K, Fretts R, Jackson LB (2021). What is good grief support? Exploring the actors and actions in social support after traumatic grief. PloS one..

[60] Rodriguez A, Spilker A, Goyal D (2020). Grief among neonatal intensive care nurses. MCN Am J Matern Child Nurs.

[61] Chirico F, Leiter M. Tackling stress, burnout, suicide and preventing the “great resignation” phenomenon among healthcare workers (during and after the COVID-19 pandemic) for maintaining the sustainability of healthcare systems and reaching the 2030 sustainable development goals. J Health Soc Sci. 2022;7(1):9–13.

[62] Lobb EA, Oldham L, Vojkovic S, Kristjanson LJ, Smith J, Brown JM (2010). Frontline grief: the workplace support needs of community palliative care nurses after the death of a patient. J Hosp Palliat Nurs.

[63] Zheng R, Lee SF, Bloomer MJ (2018). How nurses cope with patient death: A systematic review and qualitative meta-synthesis. J Clin Nurs.

[64] Povedano-Jimenez M, Granados-Gamez G, Garcia-Caro MP (2020). Work environment factors in coping with patient death among Spanish nurses: a cross-sectional survey. Rev Lat Am Enferm..

[65] Mercer DL, Evans JM (2006). The impact of multiple losses on the grieving process: an exploratory study. J Loss Trauma.

[66] Kida R, Takemura Y (2022). Working conditions and fatigue in japanese shift work nurses: a cross-sectional survey. Asian Nurs Res.

[67] Ruiz-Fernández MD, Ramos-Pichardo JD, Ibáñez-Masero O, Carmona-Rega MI, Sánchez-Ruiz MJ, Ortega-Galán ÁM (2021). Professional quality of life, self-compassion, resilience, and empathy in healthcare professionals during COVID-19 crisis in Spain. Res Nurs Health.

